# Effects of Supplementary Kelp Feeding on the Growth, Gonad Yield, and Nutritional and Organoleptic Quality of Subadult Sea Urchin (*Strongylocentrotus intermedius*) with Soya Lecithin Intake History

**DOI:** 10.1155/2023/8894923

**Published:** 2023-11-16

**Authors:** Weixiao Di, Yuqing Heqiu, Dan Gou, Panke Gong, Jun Ding, Yaqing Chang, Rantao Zuo

**Affiliations:** Key Laboratory of Mariculture and Stock Enhancement in North China's Sea (Ministry of Agriculture and Rural Affairs), Dalian Ocean University, Dalian, 116023, China

## Abstract

A 23-week feeding experiment was conducted to investigate the effects of supplementary kelp feeding on the growth, gonad development, and nutritional and sensory properties of sea urchin (*Strongylocentrotus intermedius*) with soya lecithin (SL) intake history. The feeding experiment was divided into experimental phase I and phase II. During phase I, 48 subadult sea urchins (initial weight: 6.28 ± 0.07 g) were fed one of the feeds with different levels of SL (0%, 1.6%, 3.2%) or kelp (*Saccharina japonica*) for 12 weeks. Then, all sea urchins were fed kelp for the next 11 weeks during the phase II. Each diet was randomly allocated to six cages of sea urchins. The results of phase I showed that weight gain rate (WGR), gonadosomatic index (GSI), gonad sensory properties (color and texture), and essential amino acid (EAA) contents were not significantly affected by SL level in the feed groups. High level (3.2%) of SL suppressed gonad development of *S. intermedius* with retarded gametogenesis in the 3.2% SL group (stage Ⅱ) compared to those fed 0% and 1.6% SL groups (stage Ⅲ). Sea urchins fed dry feeds exhibited significantly lower WGR and values of color (redness and yellowness) and texture (hardness and gumminess) but higher contents of EAA in the gonads than those fed kelp. The n-3/n-6 polyunsaturated fatty acid (PUFA) and eicosapentaenoic acid (EPA) of gonads in the groups fed with dry feeds showed no significant differences, but were significantly lower than that of kelp group. At the end of phase II, the gonad yellowness and EPA content of gonads in all dry feed groups were significantly increased by supplementary kelp feeding, with a higher increase observed in *S. intermedius* with SL intake history, while arachidonic acid (ARA) content was significantly improved by supplementary kelp feeding in *S. intermedius* with SL intake history. Gonad texture was improved to some extent by supplementary kelp feeding. These results indicated that *S. intermedius* fed dry feeds showed significantly higher GSI and EAA but poorer organoleptic quality and lower n-3/n-6 PUFA and EPA than those fed kelp. Kelp supplementary feeding improved the fatty acid value and organoleptic quality of gonads, especially for the sea urchins with SL intake history.

## 1. Introduction

The gonads of sea urchins also called roe are worldwide well-known for their unique texture, excellent taste, and high nutritional value, particularly in a few Asian, Mediterranean, and American nations [1, 2]. Global climate change, tight fisheries management, rapidly rising market demand, and habitat destruction [2–4] have led to a shortage of wild sea urchins. Sea urchin farming has become a good response to fill the shortage of wild resources [5]. Dalian Ocean University imported *Strongylocentrotus intermedius* from Japan to China in 1989 [6]. Since then, extensive research has been conducted on genetic breeding, aquaculture, disease prevention and control, and nutritional physiology of this species [7–11]. Nowadays, *S. intermedius* is becoming one of the most important economical farming species in northern China with an estimated annual economic worth of 100 million CNY [2, 4]. Sea urchins naturally live on fresh macroalgae, such as *Saccharina japonica* and *Undaria pinnatifida* [2]. However, the supply and nutrition of macroalgae vary all year round. This pushes forward the research and application of dry feeds that are more suitable for massive culture [4, 12, 13]. The sensory quality of sea urchin gonads is closely related to their economic values [2, 5, 14]. Although high gonad yield of sea urchins can be achieved by feeding dry feeds [2, 4, 14–16], the organoleptic quality, especially the color and texture has been reported to be poorer than those kelp produced gonads [16].

Finishing feeding with fish oil-enriched diets can successfully improve the nutritional values of the fillets of farming finfish, especially the long-chain polyunsaturated fatty acids (LC-PUFA) [17, 18]. Studies have proven that supplementary algae (e.g., *Ulva* spp. or *Gracilaria gracilis*) with dry feeds can effectively increase the color attributes of the roe of sea urchins, *Paracentrotus lividus* [19] and *Tripneustes gratilla* [20]. Soya lecithin (SL) can improve the palatability, emulsification, digestibility, absorption, transportation, and deposition of lipids. Furthermore, it was shown that the appropriate addition of SL was beneficial for the coloration of redness and yellowness in the gonads of *Strongylocentrotus purpuratus* [21] and *S. intermedius* [14]. This could be attributed to the functions of SL as good carriers of lipid-soluble vitamins and particularly carotenoids, which were positively correlated with the roe quality and nutritional value [22, 23]. Given the beneficial effects of SL on the coloration, it could be interesting for ascertaining whether the beneficial effects of SL addition on the roe quality of sea urchins can be further promoted by supplementary kelp feeding. Therefore, this study was aimed to elucidate: (i) the effects of supplementary kelp feeding on the growth, gonad yield, and nutritional and organoleptic properties of *S. intermedius* with SL intake history and (ii) whether the SL intake history enhances the effects of kelp supplementary diet on *S. intermedius*.

## 2. Materials and Methods

### 2.1. Ethics Statement

The Ethics Committee of Dalian Ocean University did not require the study to be reviewed or approved because the sea urchins are invertebrates.

### 2.2. Experimental Diets and Feeding Procedures

Kelp (*S. japonica*) (crude protein 7.98%, crude lipid 0.4%) and three isoproteinic (26%) and isolipidic (6.5%) dry feeds were used as experimental diets. The feeds were palm oil-based and formulated by including graded levels of SL (0%, 1.6%, 3.2%), which were named SL0, SL1.6, and SL3.2, respectively. The ingredients and nutrient profile of the experimental diets were shown in Tables 1 and 2. Solid ingredients were crushed and passed through a 320-*μ*m mesh. Following that, the fine powder of all solid ingredients in each feed was mixed well according to the principle of step-to-step amplification. After that, the blend of palm oil and SL oil was mixed well with the solid ingredients above. Finally, appropriately 30% water was added to the mixture above and was blended evenly. Pellet feeds (2 × 5 mm) were produced using a pelletizer (DES-TS1280, Dingrun, China). The wet feeds were spread on trays, dried in an oven at 55°C, cooled, and packed in sealed bags before they were stored at −20°C.

The feeding experiment was conducted at the experimental base of the Key Laboratory of Mariculture and Stock Enhancement in North China's Sea (Ministry of Agriculture and Rural Affairs, China). After 14 days of acclimation, 48 sea urchins of similar size (6.28 ± 0.07 g) were randomly allocated to 24 floating cages (10 cm in diameter and 20 cm in height) in a reinforced plastic tank (1,440 L) with the water flow of the rearing system maintained at an average speed of 2.0 L/min. The initial biomass was ∼0.21 g/L. Glass petri dishes (10 cm in diameter) were placed to prevent feed and feces from falling through the gaps at the bottom of the cages. Each diet was randomly assigned to six cages of sea urchins that were fed to apparent satiation at 14 : 00 daily. Every other day, the remaining feed and feces were siphoned out of the tank. The feeding experiment was comprised of two experimental phases. During experimental phase Ⅰ, sea urchins were fed one of the four experimental diets (SL0, SL1.6, SL3.2, kelp) for 12 weeks. During experimental phase Ⅱ, sea urchins in all dietary groups were fed kelp for another 11 weeks. During the feeding experiment, the following water condition was maintained: water temperature, 5–12.1°C; salinity, 30; pH, 8.0 ± 0.1; and dissolved oxygen, above 7.0 mg/L.

### 2.3. Sampling

When experimental phase Ⅰ ended, sea urchins were fasted for 24 hr and then weighed individually to calculate the weight gain rate (WGR). After that, one sea urchin was randomly selected from each cage (six individuals per treatment) and was dissected for the digestive tract and gonads. The digestive tract of each sea urchin was dissected and weighed to calculate the digestive tract index (DTI). The gonads of each sea urchin were first weighed to calculate the gonadosomatic index (GSI) and then were divided into three parts for the later analysis of histology, sensory properties (color and texture), and nutrition profile (amino acids and fatty acids). Gonads used for the analysis of sensory properties were temporarily stored at 4°C. The remaining samples were kept at −80°C after being flash-frozen by liquid nitrogen. Similar sampling procedures were adopted at the end of experimental phase Ⅱ.

### 2.4. Histological Analysis of Gonad Development

Gonadal sections were made by using the method of Santos et al. [24]. Simply, after being fixed in Bonn's reagent for 24 hr, gonad samples were dehydrated using a graded series of ethanol (75%–100%) and were then embedded in paraffin. The 4-*μ*m sections were made using a microtome, which were subsequently stained with hematoxylin and eosin dye. At last, a 40-fold light microscope (Leica, Germany) was used to observe the microstructure of gonads. The maturity stages (stages I–VI) of gonads were identified according to a study by Zhang et al. [14].

### 2.5. Gonadal Hue and Texture Analysis

Color Cue 2 Colorimeter (PANTONE, USA) was used for objective color assessment. The gonadal texture was determined using the texture analyzer (TMS-Pro, FTC, USA) [25]. A cylindrical sensor with a diameter of 20 mm was used to compress each sample twice at a rate of 60 mm/min to 50% of its initial height. Hardness (N): the maximum peak at the first compression of the specimen; springiness (mm): capacity of the specimen to rebound following the first compression; cohesiveness (ratio): internal adhesion of the specimen adhesion inside the sample; gumminess (N): the sticky properties of a semisolid specimen (hardness × cohesiveness); chewiness (mJ): the needed work of chewing a solid specimen (springiness × gumminess).

### 2.6. Nutritional Analysis

The nutritional composition of gonads was conducted according to the Association of Official Analytical Chemists (AOAC) [26]. Crude protein was determined by using the Kjeldahl method, and crude lipids were identified by Soxhlet method. Moisture content was calculated by drying the samples at 105°C to constant weight.

Fatty acids were determined by following the method described by Zuo et al. [12]. First, all fatty acids of one sample were completely methyl-esterified, and then methyl nonadecanoate (19 : 0) was mixed with the fatty acid methyl esters in each sample at a determined concentration. The fatty acid profile was determined using Thermo Fisher Trace 1310ISQ (Thermo Fisher Scientific). The temperature of the injector and detector was both set at 209°C. Following that, the temperature was raised by 10°C/min from 80 to 200°C, 5°C/min from 200 to 250°C, and 2°C/min from 250 to 270°C. Fatty acids (g/100 g) were measured based on the peak regions of the internal standards and the known quantities.

Amino acids were identified and quantified by using the method described by Luo et al. [27]. First, a sample containing 15 mg crude protein was poured into the drying hydrolysis bottle after being accurately weighed with an analytical balance. Subsequently, 10 mL hydrochloric acid (6 mol/L) was accurately added to the drying hydrolysis bottle. After blowing nitrogen with high purity nitrogen for 15 min, the bottle was immediately sealed with an alcohol torch. Then, the sample was digested in the sealed bottle at 110 ± 1°C for 22 hr and was gently shaken well after cooling. After filtration through a 0.45-*μ*m microfiltration membrane, 0.1 mL of the filtered solution was sucked into an injection bottle and then dried under high purity nitrogen gas at 60°C for 15 min. The dried sample was redissolved with 1 mL HCl (0.02 N) and was then analyzed by using a liquid chromatography (Hitachi, L8900, Japan). Amino acids were identified and quantified based on their peak duration and area in comparison to a spectrogram of external standards [28]. The absolute amino acid content was expressed as g/100 g dry sample.

According to a study by Ning et al. [2], amino acid was divided into bitter amino acid (cysteine, valine, methionine, isoleucine, leucine, tyrosine, phenylalanine, lysine, histidine, arginine); sweet amino acid (threonine, serine, glycine, alanine, proline); and umami acid (aspartate, glutamate).

### 2.7. Calculations and Statistical Analysis

According to the formulas that were reported by Ning et al. [2], the following parameters were determined.

(1) $$\begin{array}{c} \text{Survival rate }\left(\text{SR},\%\right)=N_{f}/N_{i}\times 100, \end{array}$$

(2) $$\begin{array}{c} \text{Weight growth rate }\left(\text{WGR},\%\right)=\text{(FBW-IBW)/IBW}\times 100, \end{array}$$

(3) $$\begin{array}{c} \text{Gonadosomatic index }\left(\text{GSI},\;\%\right)=\text{GW/FBW}\times 100, \end{array}$$

(4) $$\begin{array}{c} \text{Digestive tract index }\left(\text{DTI},\;\%\right)=\text{DTW/FBW}\times 100, \end{array}$$

(5) $$\begin{array}{c} \Delta E=\text{SQRT}\left[{\left(L^{*}-L^{*}s\right)}^{2}+{\left(a^{*}-a^{*}s\right)}^{2}+{\left(b^{*}-b^{*}s\right)}^{2}\right], \end{array}$$

where *N*_*i*_ and *N*_*f*_ represent the initial and final number of each sea urchin, respectively; IBW and FBW stand for the initial body weight and final body weight at each sampling; GW and DTW represent the weight of the gonad and digestive tract of sea urchin individuals at each sampling, respectively; and *L*^*∗*^, *a*^*∗*^, and *b*^*∗*^ represent the lightness, redness, and yellowness of values of the sample, respectively. The standard color of light yellow (*L*^*∗*^_*s*_ = 68.9, *a*^*∗*^_*s*_ = 28.7, *b*^*∗*^_*s*_ = 60.4) and light orange yellow (*L*^*∗*^_*s*_ = 74.6, *a*^*∗*^_*s*_ = 28.7, *b*^*∗*^_*s*_ = 66.1) was from Natural Color System cards and defined by Woods et al. [29]. Since these two colors were most favored by consumers, they were selected in this study to assess the color quality of sea urchin gonads among different dietary groups.

First, all data were checked for normality and homogeneity of variance. Then, statistical analyses were performed by using SPSS software (SPSS version 22.0). Results at the same experimental phase between different dietary groups were analyzed by one-way analysis of variance (ANOVA). When a significance was detected, Duncan's multiple range test was used to compare differences in the means between dietary treatments. Data in the same group between different phases were analyzed by using independent *t*-tests. *P* < 0.05 was considered as statistically significant. The results were expressed as mean ± standard error of the mean (SEM). The information regarding degrees of freedom within groups (*df*), statistical test *F*, is presented in the text where necessary.

## 3. Results

### 3.1. Survival, Growth, and Gonad Yield

At each experimental phase, no mortality of sea urchins was observed in all dietary groups (Table 3).

At the end of experimental phase I, sea urchins fed dry feeds showed significantly lower FBW (*df* = 20, *F* = 10.51, *P* < 0.05) and WGR (*df* = 20, *F* = 26.60, *P* < 0.05) than those fed kelp. On the contrary, sea urchins fed dry feeds showed significantly higher GW (*df* = 20, *F* = 6.49, *P* < 0.05) and GSI (*df* = 20, *F* = 1.97, *P* < 0.05) than those fed kelp. However, there were no significant differences in both growth and gonad yield of sea urchins in all the dry feed groups (*P* > 0.05). DTW of sea urchins was not significantly affected by diet category (kelp or dry feeds) or SL addition level (*P* > 0.05). However, DTI showed an increasing tendency as SL addition increased (*P* > 0.05). The DTI of sea urchins fed SL3.2 was the highest, which was comparable to that in SL0 and SL1.6 groups (*P* > 0.05) but was significantly higher than that in the kelp group (*P* < 0.05) (Table 3).

At the end of experimental phase Ⅱ, although FBW (*df* = 20, *F* = 7.14, *P* < 0.05) and WGR (*df* = 20, *F* = 8.28, *P* < 0.05) of sea urchins fed dry feeds were still significantly lower than those fed kelp, the gap between the kelp and dry feed groups was narrowed. The DTW and DTI were not significantly affected by diet category or SL addition level (*P* > 0.05). Only in the kelp group, the DTI of sea urchins in the experimental phase Ⅱ was significantly higher than that in experimental phase I (*P* < 0.05) (Table 3).

### 3.2. Gonad Development

**Figure 1:**
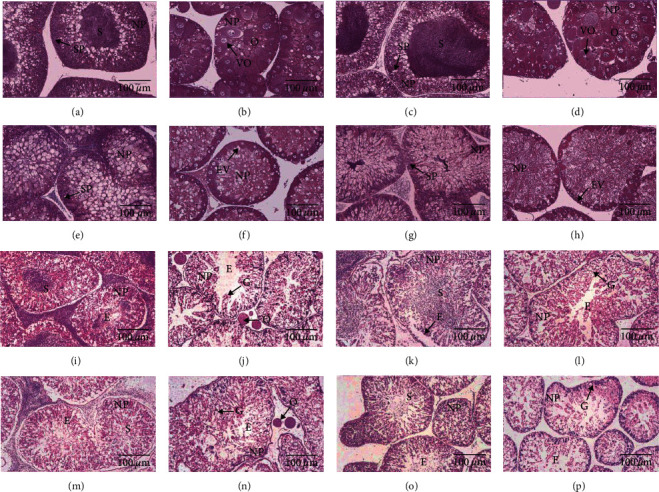
The gonad histological images of subadult sea urchin (*Strongylocentrotus intermedius*) supplemented with kelp diet and dry feed containing different levels of soya lecithin (SL). Phase Ⅰ (a–f); phase Ⅱ (i–p); (a) SL0, male, stage Ⅲ; (b) SL0, female, stage Ⅲ; (c) SL1.6, male, stage Ⅲ; (d) SL1.6, female, stage Ⅲ; (e) SL3.2, male, stage Ⅱ; (f) SL3.2, female, stage Ⅱ; (g) kelp, male, stage Ⅲ; (h) kelp, female, stage Ⅱ; (i) SL0, male, stage Ⅵ; (j) SL0, female, stage Ⅵ; (k) SL1.6, male, stage Ⅵ; (l) SL1.6, female, stage Ⅵ; (m) SL3.2, male, stage Ⅵ; (n) SL3.2, female, stage Ⅵ; (o) kelp, male, stage Ⅵ; (p) kelp, female, stage Ⅵ. SL0–SL3.2 are palm oil-based diets. NP, nutritive phagocyte; SP, spermatocyt; S, spermatozoa; EV, early vitellogenic oocyte; VO, vitellogenic oocyte; O, ovum; E, empty lumen.

At the end of the first experimental phase, male and female gonads in the SL0 and SL1.6 groups developed synchronously to stage Ⅲ, while those in the SL3.2 group still stayed at stage Ⅱ. In the kelp group, the gonads of the males developed faster than those of females, with males entering stage III while females staying at stage II (Figure 1).

At the end of the second experimental phase, both male and female gonads were synchronized to stage VI in all dietary groups (Figure 1).

### 3.3. Sensory Quality of Gonads

#### 3.3.1. Color

**Figure 2:**
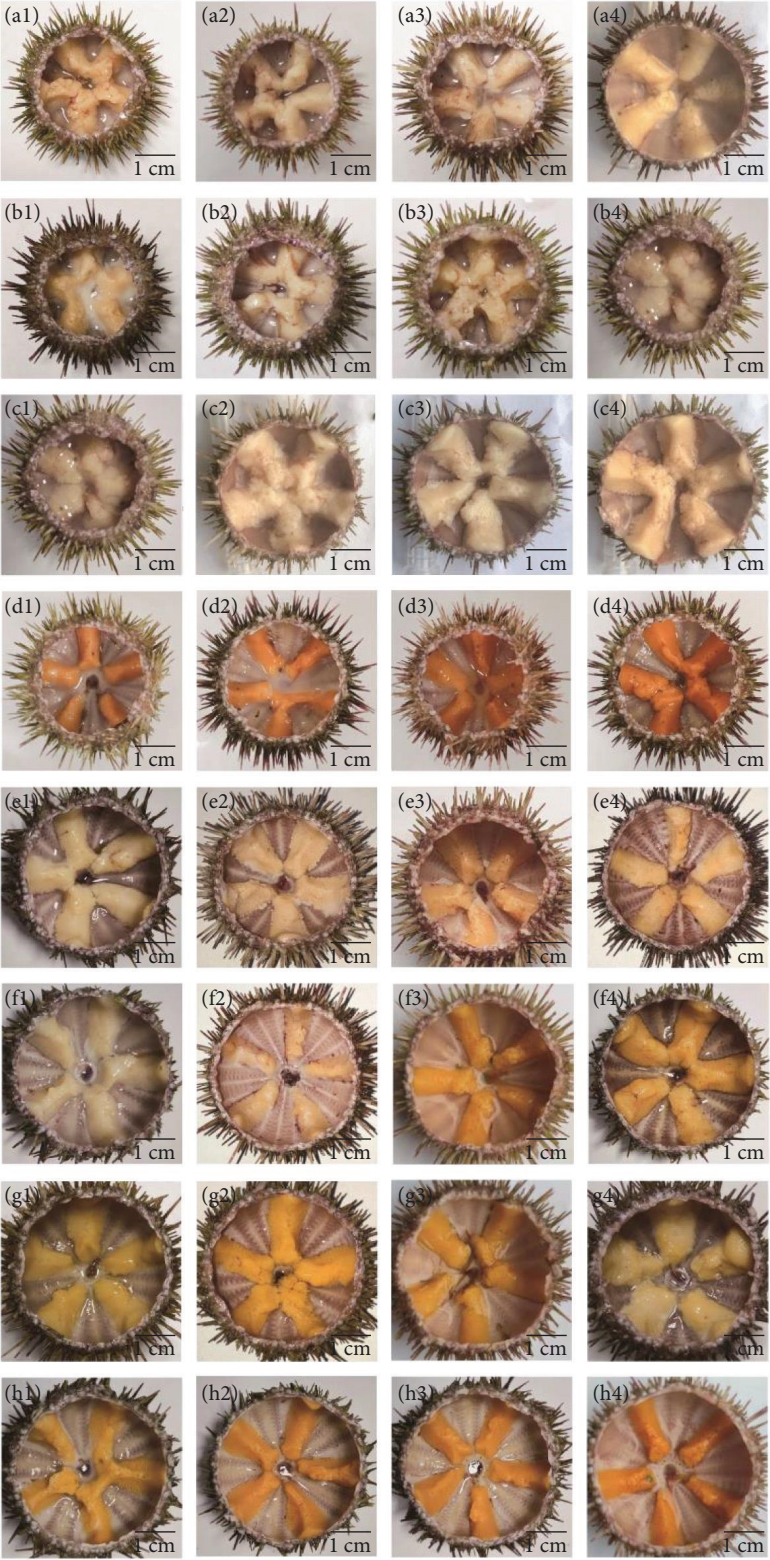
Gonad color appearance of subadult sea urchin (*Strongylocentrotus intermedius*) supplemented with kelp diet and dry feed containing different levels of soya lecithin (SL) (left male, right female). Phase Ⅰ (a–d); phase Ⅱ (e–h); (a) SL0; (b) SL1.6; (c) SL3.2; (d) kelp. SL0–SL3.2 are palm oil-based feeds.

At the end of experimental phase I, the *a*^*∗*^ (redness) (*df* = 20, *F* = 32.61, *P* < 0.05) and *b*^*∗*^ (*df* = 20, *F* = 30.16, *P* < 0.05) (yellowness) values of the kelp group were significantly higher than those of the dry feed groups (*P* < 0.05). In contrast, the *L*^*∗*^(lightness) (*df* = 20, *F* = 11.95, *P* < 0.05), *ΔE*_1_ (*df* = 20, *F* = 28.50, *P* < 0.05), and *ΔE*_2_ (*df* = 20, *F* = 25.05, *P* < 0.05) values of the kelp group were significantly lower than those of dry feed groups (Table 4). Almost all sea urchin gonads of dry feed groups were pale or light yellow (Figure 2).

The color of gonads was elevated after sea urchins were refed with kelp in all dietary groups. In all the dry feed groups, the *L*^*∗*^, *a*^*∗*^, and *b*^*∗*^values of experimental phase II were significantly higher than those of experimental phase I (*P* < 0.05). Sea urchins fed kelp showed significantly higher *L*^*∗*^ (*df* = 9, *F* = 12.58, *P* < 0.05) and *b*^*∗*^ values (*df* = 9, *F* = 0.90, *P* < 0.05) but slightly higher *a*^*∗*^ value at the end of experimental phase II (*P* > 0.05). The *ΔE*_1_ (*df* = 9, *F* = 0.50, *P* < 0.05) and *ΔE*_2_ (*df* = 9, *F* = 0.78, *P* < 0.05) values of experimental phase II were significantly lower than those of phase I for all dietary groups (Table 4). Compared to phase I, most gonads exhibited ideal color after sea urchins were supplemented with kelp (Figure 2).

At the end of experimental phase II, the *L*^*∗*^ value of the kelp group was comparable to that of the SL1.6 group (*df* = 20, *F* = 3.90, *P* > 0.05) but was significantly lower than that of the SL0 and SL3.2 groups (*P* < 0.05). The *a ^*∗*^* (*df* = 20, *F* = 17.85, *P* < 0.05) and *b ^*∗*^* (*df* = 20, *F* = 6.56, *P* < 0.05) values of the kelp group were significantly higher than that of dry feed groups. Among the feed groups, *b ^*∗*^* value of those with SL history intake was significantly higher than SL0 group (*P* < 0.05). The *ΔE*_1_ (*df* = 20, *F* = 13.24, *P* > 0.05) and *ΔE*_2_ (*df* = 20, *F* = 9.74, *P* > 0.05) values of the kelp group were significantly lower than those of the dry feed groups (*P* < 0.05). The *ΔE*_1_ and *ΔE*_2_ values of the SL1.6 group were comparable to those of the SL3.2 group (*P* < 0.05) but were significantly lower than those of the SL0 group (*P* < 0.05) (Table 4).

#### 3.3.2. Texture

At the end of experimental phase I, the hardness value of kelp group was significantly higher than that of all dry feed groups (*P* < 0.05). The springiness value of the kelp group was lowest, which was significantly lower than that of SL0 group (*P* < 0.05). On the contrary, the gumminess value of the kelp group was highest, which was significantly higher than that of SL0 (*P* < 0.05). There were no significant differences in the cohesiveness and chewiness of gonads among dietary groups (*P* > 0.05) (Table 5).

Compared to experimental phase I, the values of hardness, cohesiveness, springiness, gumminess, and chewiness of experimental phase II were elevated to different extents in the dry feed groups. The texture values of dry feed groups were higher than those of kelp group, although they were not significantly affected by SL addition level (*P* > 0.05) (Table 5).

### 3.4. Nutritional Quality of Gonads

#### 3.4.1. Proximate Composition

At the end of experimental phase I, the moisture content of SL1.6 group was highest, which was comparable to that of the kelp group (*P* > 0.05), but was significantly higher than that of SL0 and SL3.2 groups (*P* < 0.05). On the contrary, the protein content of SL1.6 group was lowest, which was comparable to that of the kelp group (*P* > 0.05), but was significantly lower than that of SL0 and SL3.2 groups (*P* < 0.05). The lipid content of kelp group was lowest, which was only significantly lower than that of SL0 group (*P* < 0.05) (Table 6).

At the end of experimental phase Ⅱ, the moisture content of SL 3.2 was significantly higher than that of other dietary groups (*P* < 0.05). The protein content of SL 3.2 was lowest, which was only significantly lower than that of SL0 group (*P* < 0.05). The lipid content of SL0 group was the highest, which was significantly higher than that of the kelp group (*P* < 0.05). The moisture content of experimental phase Ⅱ decreased while the protein content increased in all dietary groups except for SL 3.2 group compared to that of phase I (Table 6).

#### 3.4.2. Amino Acids

**Figure 3:**
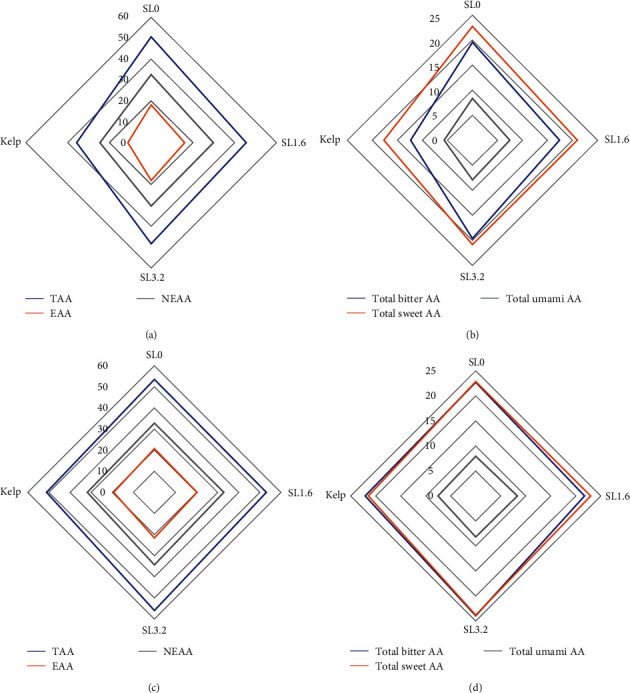
Diagram of amino acid composition (g/100 g) in the gonads of subadult sea urchin (*Strongylocentrotus intermedius*) supplemented with kelp diet and dry feed containing different levels of soya lecithin (SL). Phase Ⅰ: a and b; phase Ⅱ: c and d. SL0–SL3.2 are dry feeds with different SL addition level. TBAA, cysteine, valine, methionine, isoleucine, leucine, tyrosine, phenylalanine, lysine, histidine, and arginine; TSAA, threonine, serine, glycine, alanine, and proline; TUAA, aspartate and glutamate.

At the end of experimental phase I, sea urchins of the kelp group exhibited significantly lower contents of total amino acid (TAA), nonessential amino acid (NAA), essential amino acid (EAA), total bitter amino acid (TBAA), and total sweet amino acids (TSAA) in the gonads than those of all dry feed groups (*P* > 0.05). Similarly, the contents of total umami amino acids (TUAA) and EAA/TAA of kelp group were lowest for TUAA the significance detected between the kelp group and SL0/SL3.2 group, and for EAA/TAA the significance detected between the kelp group and SL3.2 group. However, TAA, NAA, EAA, TSAA, TUAA, and EAA/TAA were not significantly affected by SL addition level (*P* > 0.05) (*Supplementary 1*, Figure 3).

At the end of phase II, there were no significant differences in the contents of all amino acids except for aspartate (*df* = 8, *F* = 2.84, *P* > 0.05) and threonine (*df* = 8, *F* = 3.66, *P* > 0.05). The contents of TAA, EAA, and TBAA in the gonads were all increased at the end of experimental phase II than those of experimental phase I (*P* > 0.05); among them, the TAA contents in SL3.2 and kelp groups significantly increased (*P* < 0.05), the contents of EAA in kelp group significantly increased (*P* < 0.05), and the contents of TBAA in SL1.6 and kelp groups significantly increased (*P* < 0.05) (*Supplementary 1*, Figure 3).

#### 3.4.3. Fatty Acids

**Figure 4:**
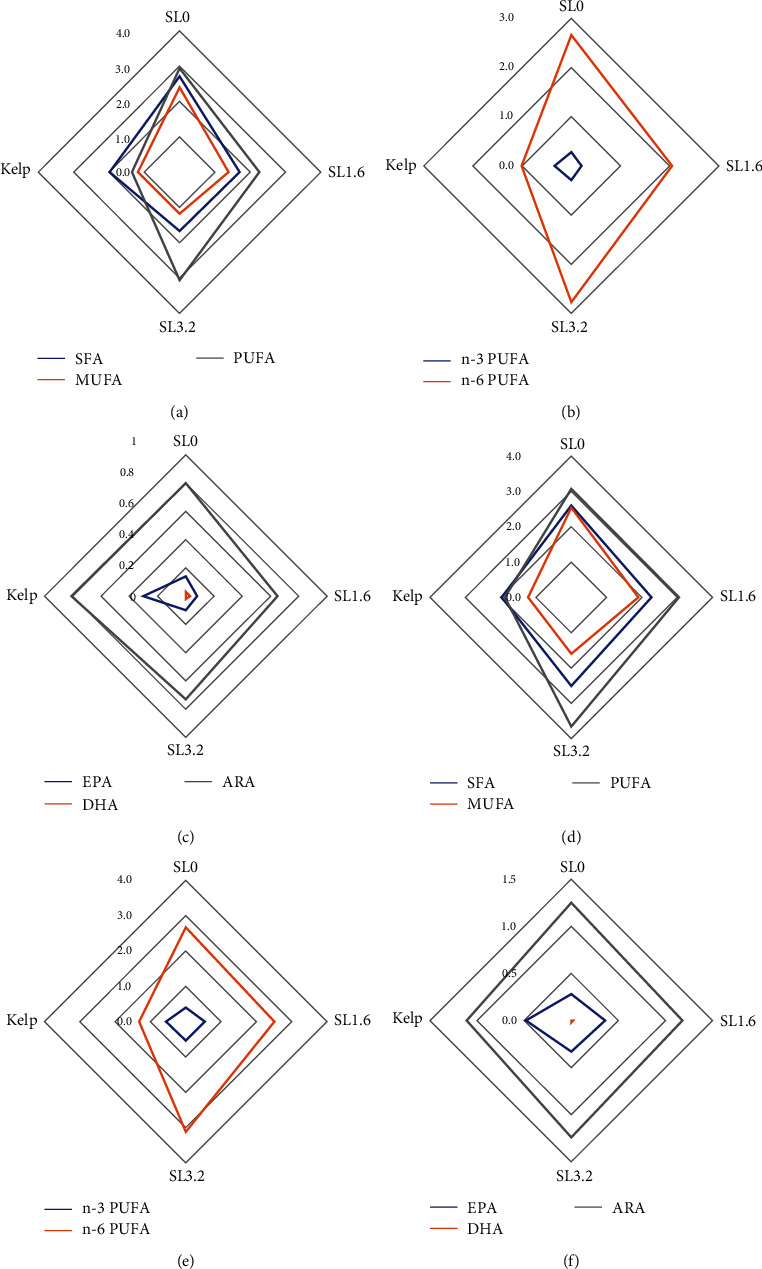
Diagram of fatty acid composition (g/100 g dry matter) in the gonads of subadult sea urchin (*Strongylocentrotus intermedius*) supplemented with kelp diet and dry feed containing different levels of soya lecithin (SL). Phase Ⅰ: a–c; phase Ⅱ: d–f. SL0–SL3.2 are dry feeds with different SL addition level. SFA, saturated fatty acids; MUFA, monounsaturated fatty acids; PUFA, polyunsaturated fatty acids; EPA, eicosapentaenoic acid (20 : 5n-3); DHA, docosahexaenoic acid (22 : 6n-3); ARA, arachidonic acid (20 : 4n-6); PUFA, polyunsaturated fatty acids.

At the end of experimental phase I, the contents of monounsaturated fatty acids (MUFA) significantly decreased as SL addition level increased in the diets (*P* < 0.05). The n-3/n-6 PUFA of gonads showed no significant differences in the dry feed groups (*df* = 8, *F* = 36.30, *P* > 0.05), which was significantly lower than that of kelp group (*P* < 0.05). Sea urchins fed kelp showed significantly higher eicosapentaenoic acid (EPA) content than those fed dry feeds (*df* = 8, *F* = 6.97, *P* < 0.05). However, the contents of EPA were not significantly affected by SL addition level in the dry feeds (*P* > 0.05) (*Supplementary 2*, Figure 4).

At the end of experimental phase II, the contents of MUFA significantly decreased as SL addition level increased in the dry feeds (*P* < 0.05). Sea urchins fed kelp showed significantly lower n-6 PUFA (*df* = 8, *F* = 13.44, *P* < 0.05) but higher n-3/n-6 PUFA than those fed dry feeds (*df* = 8, *F* = 63.95, *P* < 0.05). Different from experimental phase Ⅰ, the contents of EPA of the kelp group were comparable to SL1.6 (*df* = 3, *F* = 25.33, *P* > 0.05), which were significantly higher than those in SL0 and SL3.2 groups (*P* < 0.05). In experimental phase Ⅰ, the highest EPA was observed in SL0 among all dry feed; however, in experimental phase Ⅱ, SL0 was the lowest EPA content group among all dry feed. Compared to experimental phase I, the EPA (*df* = 4, SL0: *F* = 16.00, SL1.6: *F* = 2.57, SL3.2: *F* = 0.38, *P* < 0.05) content of all dry feed groups and arachidonic acid (ARA) (*df* = 4, SL1.6: *F* = 1.83, SL3.2: *F* = 0.73) content of SL addition feed groups were significantly increased at the end of experimental phase II (*P* < 0.05) (*Supplementary 2*, Figure 4).

## 4. Discussion

In experimental phase Ⅰ of this experiment, the weight gain ratio of *S. intermedius* in the kelp group was better than that in the feed groups. Similar results have been obtained in other sea urchin species [4, 20]. Kelp is rich in cellulose and mucus, which may be beneficial for protecting the fragile intestine and improving the digestibility of juvenile sea urchins [4]. Dietary phospholipids are considered an indispensable nutrient for several aquatic animals, especially during their larval and early juvenile stages [30]. SL is characterized by abundant phosphatidylcholine, phosphatidylethanolamine, and phosphatidylinositol [31], thus it was used as a phospholipid source widely. However, the results of this study and Zhang et al.'s study [14] found that 1.5% SL showed no beneficial effects on the growth of *S. intermedius*. Also, Gibbs et al. [30] did not find the necessity of phospholipid addition in the diets of juvenile *Lytechinus variegatus* at least from the perspective of growth performance. However, it should be noted that Gibbs et al. [30] and Zhang et al. [14] did not include a feed with SL lower than 1%. Therefore, it was still uncertain whether the beneficial effects of SL on the growth of sea urchins can be observed by a much lower addition of SL, e.g., 0.75% or even 0.5%. It is essential to quantify more precise requirements for *S. intermedius* by setting levels (may be 0%, 0.25%, 0.5%, 0.75%, 1.0%, 1.25%, and 1.5%) in the following studies.

In the experimental phase I of this study, the sea urchins fed kelp had significantly lower GSI than those fed dry feeds. This was consistent with the results of some previous studies [2, 4, 32]. Compared to dry feeds, kelp had relatively lower contents of proteins and lipids. It was found that the GSI of juvenile *S. intermedius* increased with the increasing dietary protein concentration [32]. Furthermore, insufficient lipid intake decreased gonad production of sea urchin *L. variegatus* [33]. Dietary lipid at a level of 90 g/kg achieved the maximum GSI in both male and female *Onychostoma macrolepis* broodstock [34]. Relatively higher lipid level (180 g/kg) in the diets significantly promoted gonad development of female Chinese sturgeon (*Acipenser sinensis*) [35]. Dietary ARA at a level of 1% resulted in the highest GSI of adult *S. intermediu*s [12]. The addition of 0.46%–0.89% ARA can promote the production of steroid hormones and ovarian development during the predevelopmental period of mud crab *Scylla paramamosain* [36]. High dietary n-3 LC-PUFAs promote ovarian development of silver pomfret (*Pampus argenteus*). Dietary docosahexaenoic acid (DHA)/EPA (1.09) promoted ovarian development of tongue sole (*Cynoglossus semilaevis*) [37]. In the present study, the GSI in all feed groups was subject to a reduction in experimental phase Ⅱ. On one hand, this could be due to the low contents of protein and lipid (fatty acids) of fresh large algae as mentioned above. Cyrus et al. [20] found that the GSI and gonad weight of *T.gratilla* decreased when the dry feeds were replaced by fresh *Ulva armoricana*. On the other hand, it could be due to the release of spermatozoa and ova at the end of experimental phase Ⅱ. When the samples were taken at the end of the experimental phase Ⅱ, all sea urchins were easily stimulated to spawn during daily water exchange.

At the end of experimental phase I, male and female gonads in the SL0 and SL1.6 groups developed synchronously to stage Ⅲ, while those in the SL3.2 group still stayed at stage Ⅱ. This was consistent with the findings of Zhang et al. [14] who found that gonad development can be retarded by overdose of SL. Different from egg lecithin or krill oil, SL is characterized by abundant linoleic acid (LA). Thus, increasing SL addition will unavoidably increase the retention of n-6 PUFA in the gonads. Numerous studies have proved that LA can induce inflammation and negatively impact the normal physiology of finfish [38]. Similarly, a recent study by a research team showed that SL addition induced inflammation in *S. intermediu*s [39]. Nutrition phagocytes (NPs) are somatic cells that store essential nutrients (such as protein, lipid, and glycogen) for the gametogenesis of sea urchins [40]. Excessive inflammatory response causes oxidative stress, which affects the normal physiological function of phagocytes in aquatic animals [41–44]. In this experiment, LA-induced inflammation may negatively affect the normal physiological function of NPs and gonadal development. Increasing the n-3/n-6 PUFA ratio in the dry feeds can effectively reduce the inflammation [45]. Thus, krill oil, characterized by abundant n-3 LC-PUFA, needs to be assessed for its effects on the promotion of gonad development of sea urchins in the following studies.

Color is a critical factor of determining the market value of sea urchin gonads [2]. Bright yellow, yellow-orange, and mango orange are preferred by consumers [46]. In the present study, it was found that the gonads of sea urchins fed dry feeds showed higher *L*^*∗*^ (lightness) values than those fed kelp, which was consistent with the findings of several previous studies [2, 14, 16, 20]. Pearce et al. [47] found that sea urchins fed dry feeds with starch used for the binder produced brighter gonads than those fed with other binders. It was postulated that starch induced the production of stored glycogen in the gonads, which may account for the higher lightness in all feed groups [47]. In this study, about 25% wheat meal was included in the dry feeds, which could be responsible for the pale and brighter gonads. Unlike wheat meal, corn meal containing high levels of carotenoids such as lutein, zeaxanthin, *α*-, *β*-carotene, and *β*-cryptoxanthin has been proven to exert beneficial effects on enhancing the color of egg yolks [48]. Therefore, corn meal is recommended to be used as starch source in the dry feeds of sea urchin.

Different from *L*^*∗*^ (lightness) values, the *a*^*∗*^ (redness) and *b*^*∗*^ (yellowness) values of the gonads in the dry feed groups were significantly lower than those in the kelp group. Redness and yellowness of gonads can be promoted by adding *β*-carotene, the primary category of carotenoid, to the diets of *Strongylocentrotus droebachiensis* [49], *Psammechinus miliaris* [50], and *Paracentrotus lividus* [51]. Echinenone, synthesized from the substrate *β*-carotene, is the predominate carotenoid in the gonads of most sea urchins [22, 23]. Echinenone has proved to be positively correlated with gonad color of sea urchins [50]. Since most animals cannot synthesize carotenoids *de novo* [52], diet intervention is an efficient way to accumulate carotenoids [50]. Kelp is an important source of *β*-carotene [53]. In this study, sea urchins of the dry feed groups produced gonads with markedly higher values of redness and yellowness after they were refed with kelp. This was consistent with the results of Cyrus et al. [20] who found that the redness value of the gonads of *T. gratilla* was increased when their diets were transferred from dry feeds to fresh *U. armoricana* for 12 weeks. Notably, the gonads of sea urchins with a history of SL intake exhibited significantly better coloration of yellowness compared to those deprived of SL. This indicates that SL could exert the beneficial effects by improving the absorption of carotenoids provided by kelp. It was acknowledged that phospholipids play an important role in improving the solubility and dissolution, transportation, and deposition of carotenoids [21, 54, 55]. Sex was proved to be correlated with the gonad color [22, 23, 56]. Consistently, results of this study found that the gonads of female sea urchins showed a more pleasant color than those of males after supplementary kelp diet, especially in the SL1.6 group.

The texture of the gonads is considered as important as color for appearance evaluation. Firm texture with no gonadal fluid release is the market-preferred attribute. Hardness is crucial in objectively evaluating texture [25, 57]. In this study, the hardness of gonads was affected by food category and SL addition level. In the experimental phase Ⅰ, the hardness was lower in the dry feed groups; specifically, the highest value was observed in the SL3.2 group in all feed groups. On one hand, this could be due to the slower gonadal development in the kelp and SL3.2 groups. It was commonly accepted that the texture of sea urchin gonads largely depends on the gametogenesis stage [16, 25]. This could be due to the increased protein catabolism in the nutritive phagocytes during gametogenesis [40]. On the other hand, this could be due to slower gonad yield in the kelp group. Previous studies showed that rapid gonad growth resulted in a softer texture [2, 15]. In the phase Ⅱ of this experiment, there were no significant differences in the GSI and gametogenesis stage among all dietary treatments. Consistently, the gonads showed no significant variation in the hardness. In experimental phase Ⅰ, the hardness of gonads in the kelp group was the highest, which could be due to the lowest lipid content. This was consistent with the findings of Xu et al. [58] who found that the hardness of fillets was negatively correlated with the lipid content, with tiger puffer (*Takifugu rubripes*) exhibiting the highest hardness and lowest lipid content compared to Japanese seabass (*Lateolabrax japonicus*) and turbot (*Scophthalmus maximus*). Previous studies have shown that the gonads of sea urchins have a negative correlation with moisture content [2, 15, 25]. Although such a correlation was not observed in the experimental phase Ⅰ, the correlation was existing in each feed group before and after supplementary kelp feeding.

Amino acids are categorized into three groups based on the flavors they represent: bitter amino acids, sweet amino acids, and umami amino acids [2, 16, 21]. It was found that EAA and arginine are the main contributors to bitterness. A bitter taste in gonads is one of the biggest constraints in the commercialization of sea urchin roe in the Japanese market and is usually elevated by using high protein (25%) dry feeds [21]. Consistently, results in the experimental phase I of the present study showed that the contents of TBAA in the gonads of sea urchins fed dry feeds were significantly higher than those fed kelp. After sea urchins were refed with kelp, the contents of TBAA were increased to some extent. The increased amino acids could be due to the active protein catabolism during gametogenesis. EAA in the ovarian of mud crab (*S. paramamosain*) increased significantly during initial vitellogenesis and oogenesis stage [59]. The gonad of sea urchins is highly valued for its sweet flavor in Japan [51], which is due in part to the presence of glycine. In this study, glycine was found to be the predominant amino acid in the gonads of both experimental phases. The highest glycine content was observed in the kelp group, which was consistent with the findings of previous studies on sea urchins [2, 60]. Despite the significant difference in protein content between kelp and dry feed, the higher glycine content was observed in the kelp group. In experimental phase II, the glycine content of the feed groups increased slightly. This indicated that the refeeding strategy successfully enhanced the sweet flavor of gonads of sea urchins with feed intake history.

Saturated fatty acids (SFAs) like palmitic acid (PA, 16 : 0) are abundant in the gonads of sea urchins [2, 12, 61]. SFAs are crucial sources of energy substances for sea urchins and their consumers. Since these fatty acids can be easily accessible through a variety of pathways, they are paid less attention in similar studies on marine animals. LC-PUFAs in the gonads of sea urchins are characterized by high amount of ARA (C20 : 4n-6) and EPA (C20 : 5n-3) but trace amounts of DHA (C22 : 6n-3) [2, 14]. In addition to their absolute content, the n-3/n-6 PUFA is also important for maintaining the health status of humans [62, 63]. In experimental phase Ⅰ of this study, the n-3/n-6 PUFA was significantly lower in all feed groups compared to the kelp group. This could be due to the inclusion of considerable amounts of linoleic acid (LA, 18 : 2n-6) in the palm oil and SL of feed groups [14, 64]. Since EPA comprises a considerable portion of n-3 PUFA in kelp [65], the content of EPA was highest in the kelp group, which was significantly higher than that in the feed groups. Thus, lower n-3 PUFA and high LA could be responsible for the relatively lower n-3/n-6 PUFA in the gonads of sea urchins fed dry feeds. After being supplemented with kelp for 11 weeks, sea urchin gonads showed significantly increased retention of EPA and n-3 PUFA in the feed groups. This was consistent with the findings of Ting et al. [66] who found that the darkbarbel catfish (*Pelteobagrus vachelli*) supplemented with fish oil-based feeds for 30 days improved the contents of n-3 HUFA, EPA, and DHA in the flesh of fish that were previously fed soybean oil-based feeds. Similar results showed that the contents of n-3 PUFA in European sea bass (*Dicentrarchus labrax*) with a feeding history of soybean oil-based feeds were improved after they were refed with fish oil-based feeds for 5 months [17]. However, it should be noted that supplementary with kelp did not reduce the absolute contents of LA (18 : 2n-6) in the feed groups. These results indicated that supplemented kelp enhanced the EPA content but less effectively washed out the LA.

Furthermore, it was found that a certain amount of ARA and EPA was deposited in the gonads, although there were no ARA and EPA in the dry feeds used in this study. It could be the newly synthesis of ARA and EPA that were responsible for their presence in the gonads. A recent study by our research team found that the expression of several LC-PUFA synthesis-related genes was promoted by the appropriate addition of SL [39]. Although the underlying mechanisms are not completely elucidated, it was postulated to be related to the dynamic change of steroid hormone contents during different gametogenesis stages. Steroid hormones were associated with gonadal development. It was found that the estradiol-17*β* and testosterone levels were significantly higher in the gonads of cockle (*Fulvia mutica*) during gametogenesis and spawning [67]. The highest levels of estradiol-17*β* were found in the blood of sexually mature females striped catfish (*Pangasianodon hypophthalmus*) [68]. Sexual maturation of male mice stimulated the expression of enzyme FADS1, a key rate-limiting enzyme of ARA synthesis from its substrate LA [69]. Thus, it is essential to compare the fatty acids and investigate related gene expression at different maturity stages of gonads in the following studies to better understanding the LC-PUFA synthesis-related mechanisms. Nonetheless, the possibility cannot be excluded that ARA and EPA, previously deposited in the long digestive tract of sea urchins, were transported to the gonads during their fast development when LC-PUFA was desperately needed.

To conclude, sea urchins fed dry feeds exhibited significantly lower WGR and gonad color attributes (redness and yellowness) and texture (hardness and gumminess) but higher GSI and EAA contents than those fed kelp. Supplementary kelp feeding significantly increased the yellowness and EPA content of gonads in all dry feed groups, with a higher increase observed in *S. intermedius* with SL intake history, while ARA content was significantly improved by supplementary kelp feeding in *S. intermedius* with SL intake history. Gonad texture was improved to some extent by supplementary kelp feeding although no significance was detected among dietary groups. These results indicated that *S. intermedius* fed dry feeds showed significantly higher GSI and EAA but poorer organoleptic quality and lower n-3/n-6 PUFA and EPA than those fed kelp. Kelp supplementary feeding improved the fatty acid value and organoleptic quality of gonads, especially for the sea urchins with SL intake history.

## Figures and Tables

**Table 1:** Formulation and proximate composition of the experimental diets (% dry weight).

Ingredients (%)	Dry feed treatments		
	SL0	SL1.6	SL3.2
Soybean meal^1^	10	10	10
Casein	12	12	12
Gluten^2^	10	10	10
Wheatmeal^3^	20.71	20.71	20.71
Wheat bran^4^	20	20	20
Corn starch	16	16	16
Soybean lecithin	0	1.6	3.2
Palm oil	4	2.4	0.8
Cholesterol	1	1	1
Vitamin premix^5^	2	2	2
Mineral premix^6^	2	2	2
Calcium propionate	0.18	0.18	0.18
Ethoxyquin	0.01	0.01	0.01
Choline chloride	0.1	0.1	0.1
Monocalcium phosphate	2	2	2
Proximate composition			
Crude protein	26.20	26.26	26.23
Crude lipid	6.55	6.52	6.51
* β*-Carotene	0.932	1.012	0.953

*Note*: ^1^Soybean meal: crude protein, 51.56%, crude lipid, 0.9%. ^2^Gluten: crude protein, 68.99%, crude lipid, 2.8%. ^3^Wheatmeal: crude protein, 13.88%, crude lipid, 0.6%. ^4^Wheat bran: crude protein, 19.85%; crude lipid, 4%. ^5^Vitamin premix (mg or g kg^−1^ diet): vitamin D, 5 mg; vitamin K, 10 mg; vitamin B_12_, 10 mg; vitamin B_6_, 20 mg; folic acid, 20 mg; vitamin B_1_, 25 mg; vitamin A, 32 mg; vitamin B_2_, 45 mg; pantothenic acid, 60 mg; biotin, 60 mg; niacin acid, 200 mg; *α*-tocopherol, 240 mg; inositol, 800 mg; ascorbic acid, 2,000 mg; microcrystalline cellulose, 16.47 g. ^6^Mineral premix (mg or g kg^−1^ diet): CuSO_4_ · 5H_2_O, 10 mg; Na_2_SeO_3_ (1%), 25 mg; ZnSO_4_·H_2_O, 50 mg; CoCl_2_ · 6H_2_O (1%), 50 mg; MnSO_4_·H_2_O, 60 mg; FeSO_4_·H_2_O, 80 mg; Ca(IO_3_)_2_, 180 mg; MgSO_4_ · 7H_2_O, 1,200 mg; zeolite, 18.35 g.

**Table 2:** Fatty acid profile of dry feeds (g/kg dry weight) and kelp (g/kg wet weight).

Fatty acid	Dry feed treatments			Kelp
	SL0	SL1.6	SL3.2	
C14 : 0	0.51	0.38	0.24	0.14
C16 : 0	15.99	13.26	10.31	2.01
C18 : 0	1.96	2.04	2.01	3.72
C20 : 0	0.16	0.14	0.09	—
*Σ*SFA^1^	18.62	15.82	12.65	5.87
C14 : 1	—	—	—	—
C16 : 1	0.18	0.13	0.09	0.16
C18 : 1	23.23	17.5	10.76	0.19
C20 : 1	0.18	0.15	0.14	—
C22 : 1	0.15	0.17	0.16	0.07
*Σ*MUFA^2^	23.74	17.95	11.15	0.42
C18 : 3n3	0.67	1.07	1.73	—
C20 : 3n3	—	—	—	—
C20 : 5n3	—	—	—	0.08
C22 : 6n3	—	—	—	—
*Σ*n-3 PUFA^3^	0.79	1.07	1.73	0.08
C18 : 2n-6	15.03	16.74	20.04	0.10
C18 : 3n6	—	—	—	0.04
C20 : 3n6	—	—	—	—
C20 : 4n6	—	—	—	0.16
*Σ*n-6 PUFA	15.07	16.74	20.04	0.27
n-3/n-6 PUFA	0.05	0.06	0.09	0.26
ARA/EPA	—	—	—	2.00
DHA/EPA	—	—	—	—

*Note*: “—” means not detected. ^1^SFA, saturated fatty acids. ^2^MUFA, monounsaturated fatty acids. ^3^PUFA, polyunsaturated fatty acids.

**Table 3:** Survival and growth performance of subadult sea urchin (*Strongylocentrotus intermedius*) supplemented with kelp diet and dry feed containing different levels of soya lecithin (SL)^1^.

	Phase Ⅰ				Phase Ⅱ			
	Kelp	SL0	SL1.6	SL3.2	Kelp	SL0	SL1.6	SL3.2
SR (%)^2^	100 ± 0.00	100 ± 0.00	100 ± 0.00	100 ± 0.00	100 ± 0.00	100 ± 0.00	100 ± 0.00	100 ± 0.00
IBW (g)^3^	6.07 ± 0.30	6.11 ± 0.19	6.53 ± 0.14	6.19 ± 0.15				
FBW (g)^3^	18.28 ± 0.62^a^ ^*∗*^	14.07 ± 0.56^b^ ^*∗*^	13.24 ± 0.76^b^ ^*∗*^	13.48 ± 0.77^b^ ^*∗*^	26.78 ± 1.84^a^ ^*∗*^	21.26 ± 1.30^b^ ^*∗*^	20.38 ± 1.59^b^ ^*∗*^	17.32 ± 1.05^*∗*^^b^
WGR (%)^4^	202.6 ± 7.1^a^ ^*∗*^	114.8 ± 10.1^b^ ^*∗*^	104.4 ± 7.5^b^ ^*∗*^	116.8 ± 7.6^b^ ^*∗*^	331.7 ± 39.7^a^ ^*∗*^	235.5 ± 10.0^b^ ^*∗*^	208.7 ± 19.7^b^ ^*∗*^	174.3 ± 11.9^b^ ^*∗*^
GW (g)^5^	0.92 ± 0.16^b^ ^*∗*^	1.84 ± 0.20^a^	2.02 ± 0.17^a^ ^*∗*^	1.58 ± 0.22^a^	1.47 ± 0.14^*∗*^	1.62 ± 0.15	1.25 ± 0.16^*∗*^	1.33 ± 0.12
GSI (%)^6^	5.10 ± 0.88^b^	14.47 ± 1.37^a^ ^*∗*^	15.47 ± 1.47^a^ ^*∗*^	12.07 ± 2.06^a^	5.92 ± 0.74	7.65 ± 0.64^*∗*^	6.11 ± 0.53^*∗*^	7.85 ± 0.90
DTW (g)^7^	0.51 ± 0.04^*∗*^	0.46 ± 0.03^*∗*^	0.47 ± 0.05^*∗*^	0.51 ± 0.06^*∗*^	1.17 ± 0.14^*∗*^	0.94 ± 0.12^*∗*^	0.83 ± 0.12^*∗*^	0.83 ± 0.09^*∗*^
DTI (%)^8^	2.78 ± 0.26^b^ ^*∗*^	3.30 ± 0.29^ab^	3.49 ± 0.23^ab^	3.77 ± 0.31^a^	4.32 ± 0.29^*∗*^	4.37 ± 0.39	3.98 ± 0.38	4.84 ± 0.44

*Note*: ^1^Means with different superscript lowercase letters in the same row indicate significant differences between different dietary groups in the same period at *P* < 0.05. Means with superscript “ ^*∗*^” indicate significant differences between different periods in the same group at *P* < 0.05. ^2^SR, survival rate. ^3^IBW, initial body weight. FBW, final body weight. ^4^WGR, weight gain rate. ^5^GW, gonad weight. ^6^GSI, gonadosomatic index. ^7^DTW, digestive tract weight. ^8^DTI, digestive tract index.

**Table 4:** Gonad color of subadult sea urchins (*Strongylocentrotus intermedius*) supplemented with kelp diet and dry feed containing different levels of soya lecithin (SL)^1^.

	Phase Ⅰ				Phase Ⅱ			
	Kelp	SL0	SL1.6	SL3.2	Kelp	SL0	SL1.6	SL3.2
*L* ^*∗*^^2^	57.10 ± 0.28^b^ ^*∗*^	65.32 ± 0.98^a^ ^*∗*^	62.97 ± 1.38^a^ ^*∗*^	65.49 ± 1.18^a^ ^*∗*^	71.50 ± 2.26^b^ ^*∗*^	78.99 ± 1.50^a^ ^*∗*^	76.18 ± 1.52^ab^ ^*∗*^	77.33 ± 0.96^a^ ^*∗*^
*a* ^*∗*^^3^	19.93 ± 1.54^a^	7.58 ± 0.96^b^ ^*∗*^	6.49 ± 0.88^b^ ^*∗*^	7.59 ± 0.93^b^ ^*∗*^	24.90 ± 2.11^a^	11.29 ± 0.68^b^ ^*∗*^	14.85 ± 1.04^b^ ^*∗*^	14.38 ± 1.36^b^ ^*∗*^
*b* ^*∗*^^4^	37.27 ± 1.07^a^ ^*∗*^	20.86 ± 1.36^b^ ^*∗*^	18.85 ± 1.46^b^ ^*∗*^	20.92 ± 1.83^b^ ^*∗*^	48.98 ± 2.22^a^ ^*∗*^	36.76 ± 1.30^b^ ^*∗*^	43.73 ± 2.86^a^ ^*∗*^	43.57 ± 2.86^a^ ^*∗*^
*Δ*E1^5^	27.52 ± 1.42^b^ ^*∗*^	45.04 ± 1.52^a^ ^*∗*^	47.61 ± 1.59^a^ ^*∗*^	45.00 ± 1.94^a^ ^*∗*^	14.22 ± 2.10^c^ ^*∗*^	31.29 ± 1.16^a^ ^*∗*^	23.04 ± 1.49^b^ ^*∗*^	24.09 ± 2.60^b^ ^*∗*^
*Δ*E2^6^	34.96 ± 1.32^b^ ^*∗*^	50.86 ± 1.46^a^ ^*∗*^	53.59 ± 1.61^a^ ^*∗*^	50.79 ± 1.92^a^ ^*∗*^	19.06 ± 2.30^c^ ^*∗*^	34.61 ± 1.21^a^ ^*∗*^	26.59 ± 1.17^b^ ^*∗*^	27.07 ± 2.91^b^ ^*∗*^

*Note*: ^1^Means with different superscript lowercase letters in the same row indicate significant differences between different dietary groups in the same period at *P* < 0.05. Means with superscript “ ^*∗*^” indicate significant differences between different periods in the same group at *P* < 0.05. ^2^*L*^*∗*^, lightness. ^3^*a ^*∗*^*, redness. ^4^*b*^*∗*^, yellowness. ^5^*ΔE*_1_, average chromatic aberration, compared with standard light orange-yellow. ^6^*ΔE*_2_, average chromatic aberration, compared with standard light yellow. The lower the value of *ΔE*, the greater the similarity with the standard color.

**Table 5:** Gonad texture profile analysis (TPA) of subadult sea urchins (*Strongylocentrotus intermedius*) supplemented with kelp diet and dry feed containing different levels of soya lecithin (SL)^1^.

	Phase Ⅰ				Phase Ⅱ			
	Kelp	SL0	SL1.6	SL3.2	Kelp	SL0	SL1.6	SL3.2
Hardness	0.90 ± 0.04^a^	0.73 ± 0.02^b^ ^*∗*^	0.70 ± 0.03^b^ ^*∗*^	0.77 ± 0.02^b^	0.89 ± 0.05	0.96 ± 0.06^*∗*^	0.83 ± 0.04^*∗*^	0.86 ± 0.06
Cohesiveness	0.24 ± 0.02	0.23 ± 0.01^*∗*^	0.25 ± 0.02	0.22 ± 0.01^*∗*^	0.24 ± 0.02^b^	0.32 ± 0.02^a^ ^*∗*^	0.29 ± 0.02^ab^	0.30 ± 0.01^a^ ^*∗*^
Springiness	0.15 ± 0.01^b^ ^*∗*^	0.19 ± 0.01^a^	0.18 ± 0.01^ab^ ^*∗*^	0.16 ± 0.01^b^ ^*∗*^	0.20 ± 0.01^*∗*^	0.22 ± 0.02	0.23 ± 0.02^*∗*^	0.20 ± 0.01^*∗*^
Gumminess	0.22 ± 0.03^a^	0.17 ± 0.01^b^ ^*∗*^	0.18 ± 0.01^ab^ ^*∗*^	0.17 ± 0.01^ab^ ^*∗*^	0.22 ± 0.02^b^	0.31 ± 0.03^a^ ^*∗*^	0.24 ± 0.03^ab^ ^*∗*^	0.26 ± 0.02^ab^ ^*∗*^
Chewiness	0.04 ± 0.00	0.03 ± 0.00^*∗*^	0.03 ± 0.00^*∗*^	0.03 ± 0.00^*∗*^	0.04 ± 0.01	0.07 ± 0.01^*∗*^	0.06 ± 0.01^*∗*^	0.05 ± 0.00^*∗*^

*Note*. ^1^Means with different superscript lowercase letters in the same row indicate significant differences between different dietary groups in the same period at *P* < 0.05. Means with superscript “ ^*∗*^” indicate significant differences between different periods in the same group at *P* < 0.05.

**Table 6:** Proximate composition (% wet weight) in the gonads of subadult sea urchin (*Strongylocentrotus intermedius*) supplemented with kelp diet and dry feed containing different levels of soya lecithin (SL)^1^.

	Phase Ⅰ				Phase Ⅱ			
	Kelp	SL0	SL1.6	SL3.2	Kelp	SL0	SL1.6	SL3.2
Moisture	77.68 ± 0.85^ab^	74.33 ± 1.50^b^	78.70 ± 0.94^a^ ^*∗*^	74.70 ± 0.28^b^	75.00 ± 0.70^b^	73.43 ± 0.55^b^	75.47 ± 0.19^b^ ^*∗*^	78.20 ± 1.10^a^
Crude protein	11.21 ± 0.4^b^ ^*∗*^	13.20 ± 0.8^a^	11.01 ± 0.5^b^ ^*∗*^	13.13 ± 0.1^a^ ^*∗*^	13.58 ± 0.6^ab^ ^*∗*^	14.4 ± 0.6^a^	13.6 ± 0.01^ab^ ^*∗*^	12.2 ± 0.1^b^ ^*∗*^
Crude lipid	2.02 ± 0.39^b^	4.15 ± 0.43^a^	2.33 ± 0.30^b^	3.18 ± 0.68^ab^	2.18 ± 0.39^b^	3.66 ± 0.43^a^	3.00 ± 0.30^ab^	3.03 ± 0.68^ab^

*Note*. ^1^Means with different superscript lowercase letters in the same row indicate significant differences between different dietary groups in the same period at *P* < 0.05. Means with superscript “ ^*∗*^” indicate significant differences between different periods in the same group at *P* < 0.05.

## Data Availability

The data of this study can be provided by the corresponding author on reasonable request.

## Supplementary Materials

Supplementary 1 Amino acid profile (g/100 g dry weight) in the gonads of subadult sea urchins (*Strongylocentrotus intermedius*) supplemented with kelp diet and dry feed containing different levels of soya lecithin (SL)^1^.

Supplementary 2 Fatty acid composition (g/100 g dry gonad weight) of subadult sea urchins (*Strongylocentrotus intermedius*) supplemented with kelp diet and dry feed containing different levels of soya lecithin (SL)^1^.
