# Supplementary material for: Effects of Supplementary Kelp Feeding on the Growth, Gonad Yield, and Nutritional and Organoleptic Quality of Subadult Sea Urchin (Strongylocentrotus intermedius) with Soya Lecithin Intake History
Source: Aquac Nutr. 2023 Nov 16;2023:8894923. doi: 10.1155/2023/8894923 (PMC10667049; doi:10.1155/2023/8894923)
Supplement: Supplementary 1 — Amino acid profile (g/100 g dry weight) in the gonads of subadult sea urchins (Strongylocentrotus intermedius) supplemented with kelp diet and dry feed containing different levels of soya lecithin (SL)1. [file 8894923.f1.docx]

**Supplementary Table 2:** Fatty acid composition (g/100g dry gonad weight) of subadult sea urchins (*Strongylocentrotus intermedius*) supplemented with kelp diet and dry feed containing different levels of soya lecithin (SL) ^1^.

|  | Phase Ⅰ | | | |  | Phase Ⅱ | | | |
| --- | --- | --- | --- | --- | --- | --- | --- | --- | --- |
|  | Kelp | SL0 | SL1.6 | SL3.2 |  | Kelp | SL0 | SL1.6 | SL3.2 |
| C14:0 | 0.53±0.19 | 0.49±0.06 | 0.28±0.06 | 0.22±0.04^*^ |  | 0.44±0.06 | 0.43±0.08 | 0.49±0.07 | 0.52±0.01^*^ |
| C16:0 | 1.42±0.30 | 2.18±0.19 | 1.39±0.14 | 1.42±0.28 | | 1.48±0.16 | 2.09±0.27 | 1.72±0.20 | 1.93±0.12 |
| C20:0 | 0.04±0.01 | 0.03±0.00^*^ | 0.03±0.00 | 0.03±0.01^*^ | | 0.05±0.00 | 0.08±0.01^*^ | 0.05±0.01 | 0.06±0.00^*^ |
| ∑SFA^2^ | 1.98±0.49 | 2.71±0.25 | 1.70±0.20 | 1.67±0.31 | | 1.97±0.22 | 2.60±0.34 | 2.27±0.27 | 2.51±0.12 |
| C14:1 | 0.02±0.02 | 0.02±0.00 | 0.00±0.00^*^ | 0.02±0.02 | | 0.03±0.01^a^ | 0.00±0.00^b^ | 0.04±0.00^a*^ | 0.02±0.00^ab^ |
| C16:1 | 0.26±0.07 | 0.25±0.03^*^ | 0.17±0.02^*^ | 0.16±0.04^*^ | | 0.31±0.07 | 0.43±0.02^*^ | 0.37±0.07^*^ | 0.31±0.01^*^ |
| C18:1 | 0.44±0.17^b^ | 1.41±0.24^a^ | 0.76±0.19^ab^ | 0.63±0.20^b^ | | 0.40±0.02^c^ | 1.41±0.13^a^ | 1.03±0.06^b^ | 0.80±0.06^b^ |
| C20:1 | 0.46±0.10^ab^ | 0.71±0.08^a^ | 0.46±0.08^ab^ | 0.38±0.02^b*^ | | 0.47±0.01^b^ | 0.70±0.10^a^ | 0.47±0.05^b^ | 0.48±0.01^b*^ |
| ∑MUFA^3^ | 1.18±0.36^b^ | 2.40±0.28^a^ | 1.39±0.16^b^ | 1.18±0.24^b^ | | 1.21±0.10^c^ | 2.54±0.23^a^ | 1.90±0.15^b^ | 1.60±0.07^bc^ |
| C18:3n-3 | 0.05±0.02^b^ | 0.11±0.01^ab^ | 0.11±0.01^ab^ | 0.17±0.05^a^ | | 0.09±0.00^c^ | 0.12±0.01^bc^ | 0.16±0.02^ab^ | 0.20±0.02^a^ |
| C20:5n-3 | 0.29±0.04^a*^ | 0.14±0.01^b*^ | 0.08±0.03^b*^ | 0.10±0.02^b*^ | | 0.48±0.03^a*^ | 0.28±0.04^b*^ | 0.36±0.05^ab*^ | 0.33±0.03^b*^ |
| C22:6n-3 | - | 0.04±0.01 | 0.02±0.01 | 0.02±0.00 | | - | - | 0.02±0.00 | 0.02±0.00 |
| ∑n-3PUFA^4^ | 0.34±0.06^*^ | 0.28±0.02 | 0.21±0.03^*^ | 0.29±0.04^*^ | | 0.56±0.03^a*^ | 0.40±0.04^b^ | 0.54±0.07^ab*^ | 0.54±0.01^ab*^ |
| C18:2n-6 | 0.20±0.06^b^ | 1.86±0.33^a^ | 1.59±0.28^a^ | 2.05±0.62^a^ | | 0.21±0.01^c^ | 1.41±0.16^ab^ | 1.33±0.07^b^ | 1.89±0.24^a^ |
| C20:4n-6 | 0.81±0.13 | 0.80±0.07 | 0.65±0.07^*^ | 0.73±0.08^*^ | | 1.11±0.06 | 1.25±0.18 | 1.18±0.12^*^ | 1.24±0.05^*^ |
| ∑n-6PUFA | 1.01±0.19^b^ | 2.66±0.37^a^ | 2.05±0.32^a^ | 2.08±0.20^a*^ | | 1.32±0.06^b^ | 2.66±0.34^a^ | 2.51±0.14^a^ | 3.12±0.20^a*^ |
| n-3/n-6PUFA | 0.34±0.01^a*^ | 0.11±0.02^b^ | 0.11±0.03^b*^ | 0.11±0.02^b*^ | | 0.42±0.01^a*^ | 0.15±0.02^c^ | 0.21±0.02^b*^ | 0.18±0.02^bc*^ |

^1^ Means with different superscript lowercase letters in the same row indicate significant differences between different dietary groups in the same period at *P* < 0.05. Means with superscript “*” indicate significant differences between different periods in the same group at *P* < 0.05, and “-” means not detected.

^2^SFA, Saturated fatty acids; ^3^MUFA, Monounsaturated fatty acids; ^4^PUFA, Polyunsaturated fatty acids
