# Supplementary material for: Effects of Supplementary Kelp Feeding on the Growth, Gonad Yield, and Nutritional and Organoleptic Quality of Subadult Sea Urchin (Strongylocentrotus intermedius) with Soya Lecithin Intake History
Source: Aquac Nutr. 2023 Nov 16;2023:8894923. doi: 10.1155/2023/8894923 (PMC10667049; doi:10.1155/2023/8894923)
Supplement: Supplementary 2 — Fatty acid composition (g/100 g dry gonad weight) of subadult sea urchins (Strongylocentrotus intermedius) supplemented with kelp diet and dry feed containing different levels of soya lecithin (SL)1. [file 8894923.f2.docx]

**Supplementary Table 1:** Amino acid profile (g/100g dry weight) in the gonads of subadult sea urchins (*Strongylocentrotus intermedius*) supplemented with kelp diet and dry feed containing different levels of soya lecithin (SL) ^1^.

|  | Phase Ⅰ | | | |  | Phase Ⅱ | | | |
| --- | --- | --- | --- | --- | --- | --- | --- | --- | --- |
|  | Kelp | SL0 | SL1.6 | SL3.2 |  | Kelp | SL0 | SL1.6 | SL3.2 |
| Asp^Δ^ | 2.07±0.48^b^ | 3.31±0.37^a^ | 3.00±0.22^ab^ | 3.03±0.22^ab^ |  | 3.05±0.10^b^ | 3.46±0.07^ab^ | 3.68±0.28^a^ | 3.50±0.08^ab^ |
| Thr^▽^ | 1.95±0.40^b*^ | 3.08±0.26^a^ | 2.80±0.15^ab*^ | 2.89±0.21^a*^ |  | 3.15±0.09^b*^ | 3.49±0.03^ab^ | 3.77±0.19^a*^ | 3.78±0.23^a*^ |
| Ser^▽^ | 2.27±0.35^b*^ | 4.40±0.30^a^ | 3.87±0.16^a^ | 3.93±0.34^a^ |  | 3.79±0.02^*^ | 4.31±0.27 | 4.23±0.35 | 4.45±0.40 |
| Glu^Δ^ | 3.58±0.52^b^ | 5.04±0.22^a^ | 4.48±0.14^ab^ | 4.88±0.41^a^ |  | 4.50±0.15 | 4.47±0.14 | 4.66±0.38 | 4.68±0.09 |
| Gly^▽^ | 8.55±0.82 | 8.50±0.27 | 7.88±0.08 | 7.50±0.38 |  | 8.29±0.24 | 8.82±0.13 | 8.57±0.62 | 8.84±0.44 |
| Ala^▽^ | 2.61±0.24^c^ | 4.22±0.15^a^ | 3.68±0.15^ab^ | 3.53±0.24^b^ |  | 3.44±0.06 | 3.58±0.22 | 3.57±0.35 | 3.67±0.23 |
| Cys^□^ | 0.16±0.03 | 0.22±0.02 | 0.20±0.02 | 0.19±0.02 |  | 0.20±0.01 | 0.20±0.04 | 0.25±0.01 | 0.23±0.01 |
| Val^□^ | 1.13±0.24^b*^ | 2.00±0.06^a*^ | 1.91±0.03^a*^ | 2.00±0.20^a^ |  | 2.28±0.06^*^ | 2.45±0.07^*^ | 2.29±0.05^*^ | 2.45±0.12 |
| Met^□^ | 0.55±0.20^*^ | 0.93±0.07 | 0.89±0.11^*^ | 0.90±0.10 |  | 1.13±0.02^*^ | 1.16±0.16 | 1.26±0.07^*^ | 1.24±0.12 |
| Ile^□^ | 1.41±9.17 | 2.12±0.17 | 1.95±0.10^*^ | 2.26±0.34 |  | 2.28±0.05 | 2.61±0.13 | 2.52±0.17^*^ | 2.59±0.29 |
| Leu^□^ | 2.17±0.38^b*^ | 3.64±0.16^a^ | 3.30±0.04^a*^ | 3.75±0.49^a^ |  | 3.83±0.06^*^ | 4.18±0.12 | 4.03±0.17^*^ | 4.19±0.14 |
| Tyr^□^ | 0.81±0.20^b*^ | 1.61±0.18^a^ | 1.58±0.11^ab^ | 1.68±0.38^a^ |  | 1.83±0.07^*^ | 1.97±0.14 | 1.80±0.12 | 2.04±0.20 |
| Phe^□^ | 1.23±0.20^b^ | 1.66±0.01^ab*^ | 1.49±0.07^ab*^ | 1.85±0.18^a^ |  | 1.67±0.12 | 1.98±0.02^*^ | 1.88±0.09^*^ | 1.96±0.13 |
| Lys^□^ | 1.89±0.31^*^ | 2.74±0.34 | 2.21±0.21 | 2.75±0.20 |  | 3.61±0.04^*^ | 3.21±0.49 | 2.92±0.38 | 3.68±0.45 |
| His^□^ | 0.79±0.21^b^ | 1.89±0.03^a^ | 1.50±0.13^a^ | 1.68±0.19^a^ |  | 1.34±0.09 | 1.65±0.17 | 1.53±0.08 | 1.70±0.21 |
| Arg^□^ | 2.21±0.18^*^ | 2.74±0.27 | 2.38±0.21 | 2.55±0.10 |  | 3.94±0.10^*^ | 3.31±0.26 | 3.29±0.33 | 3.87±0.55 |
| Pro^▽^ | 2.31±0.17^b*^ | 2.55±0.25^ab^ | 2.74±0.16^ab^ | 2.98±0.04^a^ |  | 2.78±0.02^*^ | 2.66±0.04 | 2.84±0.22 | 3.05±0.05 |
| TAA^2^ | 35.69±4.32^b*^ | 50.65±1.76^a^ | 45.85±0.48^a^ | 48.38±1.95^a*^ |  | 51.11±0.75^*^ | 53.50±2.12 | 53.08±3.13 | 55.93±1.66^*^ |
| EAA^3^ | 11.13±2.24^b*^ | 18.05±0.62^a^ | 16.04±0.13^a^ | 18.09±1.47^a^ |  | 19.29±0.35^*^ | 20.73±1.12 | 20.21±1.00 | 21.59±0.63 |
| NAA^4^ | 24.56±2.17^b^ | 32.60±1.14^a^ | 29.81±0.45^a^ | 30.29±0.91^a*^ |  | 31.82±0.41 | 32.78±1.03 | 32.88±2.14 | 34.34±1.12^*^ |
| TBAA^5^ | 12.35±2.18^b*^ | 19.55±0.48^a^ | 17.41±0.49^a*^ | 19.63±1.57^a^ |  | 22.11±0.31^*^ | 22.71±1.48 | 21.77±1.05^*^ | 23.94±0.80 |
| TSAA^6^ | 17.69±1.42^b^ | 22 .75±0.93^a^ | 20.97±0.37^a^ | 20.84±0.61^a^ |  | 21.45±0.27 | 22.86±0.60 | 22.98±1.67 | 23.80±0.89 |
| TUAA^7^ | 5.66±1.00^b^ | 8.35±0.53^a^ | 7.48±0.17^ab^ | 7.91±0.53^a^ |  | 7.55±0.21 | 7.93±0.21 | 8.33±0.64 | 8.19±0.05 |
| EAA/TAA | 0.31±0.03^b^ | 0.36±0.00^ab*^ | 0.35±0.01^ab*^ | 0.37±0.02^a^ |  | 0.38±0.00 | 0.39±0.01^*^ | 0.38±0.01^*^ | 0.39±0.00 |

^1^ Means with different superscript lowercase letters in the same row indicate significant differences between different dietary groups in the same period at *P* < 0.05. Means with superscript “*” indicate significant differences between different periods in the same group at *P* < 0.05.

^2^TAA: total amino acids; ^3^EAA: essential amino acids; ^4^NAA: non-essential amino acids; ^5^TBAA(□): Total bitter amino acids; ^6^TSAA(▽): Total sweet amino acids; ^7^TUAA(∆):Total umami amino acids.
